# Association of Co-dominant Immunoglobulin G Deposit in Immunoglobulin A Nephropathy With Poor Clinicopathological and Laboratory Parameters

**DOI:** 10.7759/cureus.15813

**Published:** 2021-06-21

**Authors:** Pranjal Kalita, Jaya Mishra, Biswajit Dey, Himesh Barman, Monaliza Lyngdoh

**Affiliations:** 1 Pathology, North Eastern Indira Gandhi Regional Institute of Health and Medical Sciences (NEIGRIHMS), Shillong, IND; 2 Paediatrics, North Eastern Indira Gandhi Regional Institute of Health and Medical Sciences (NEIGRIHMS), Shillong, IND; 3 Internal Medicine, North Eastern Indira Gandhi Regional Institute of Health and Medical Sciences (NEIGRIHMS), Shillong, IND

**Keywords:** iga nephropathy, immunofluorescence, igg, creatinine, proteinuria

## Abstract

Introduction: Immunoglobulin A (IgA) nephropathy is the commonest primary glomerulonephritis with a wide range of clinical presentations and laboratory findings. There is a paucity of studies regarding the significance of co-dominant immunoglobulin G (IgG) deposition in IgA nephropathy.

Methods: The study included retrospective and prospective biopsy-proven cases of IgA nephropathy from 2013-2020 with a minimum of eight glomeruli. Clinical and laboratory parameters were analysed for the IgA and IgG co-dominant cases as compared to those of the non-IgG group.

Results: A total of 58 cases of IgA nephropathy were included in the study out of which 25 biopsies (43.1%) were categorized as IgA plus IgG, and the rest 33 biopsies (56.8%) as the non-IgG group. A significant correlation was noted amongst the IgA plus IgG group with respect to the elevated mean arterial pressure (MAP) (p=0.038) and proteinuria (p=0.002) as compared to the non-IgG group. Amongst the MEST-C (mesangial hypercellularity, endocapillary hypercellularity, segmental sclerosis, tubular atrophy/interstitial fibrosis, crescents) variables, endocapillary hypercellularity correlated with elevated MAP (p=0.04), raised serum creatinine (p=0.005), and decreased estimated glomerular filtration rate (eGFR) (p=0.002).

Conclusion: Co-dominant IgG deposit serves as an adverse marker pointing towards a deranged renal function in IgA nephropathy.

## Introduction

Immunoglobulin A (IgA) nephropathy characterized by the presence of dominant or co-dominant mesangial IgA immune deposits, often accompanied by complement component 3 (C3) and immunoglobulin G (IgG) in association with proliferative glomerulonephritis of varying severity, was first described by Berger and Hinglais in 1968 [1,2]. Nowadays, it has assumed increasing importance with more and more cases being diagnosed with the advent of immunofluorescence. Globally, it is the commonest form of primary glomerulopathy [2]. The prevalence of IgA nephropathy is noted to be highest in East and Pacific Asian countries with Japan reporting >30% of adult patients and >20% of children with chronic glomerulonephritis to have this disease [3].

IgG deposits are not essential to the definitive diagnosis of IgA nephropathy, so little attention has been paid to the clinical significance of IgG deposition [3]. Moreover, the new Oxford MEST-C (mesangial hypercellularity, endocapillary hypercellularity, segmental sclerosis, tubular atrophy/interstitial fibrosis, crescents) scoring system 2016 does not include immunostains, although the location of glomerular IgA along with IgG immune deposits demonstrated by immunofluorescence studies correlates with mesangial and endocapillary hypercellularity, which according to the new MEST-C score are strong prognostic indicators in IgA nephropathy patients [4]. Animal model studies have demonstrated that mesangial IgG deposition was associated with greater inflammation [5].

The present study was undertaken with the aim to study the significance of IgG in IgA nephropathy.

## Materials and methods

**Figure 1:**
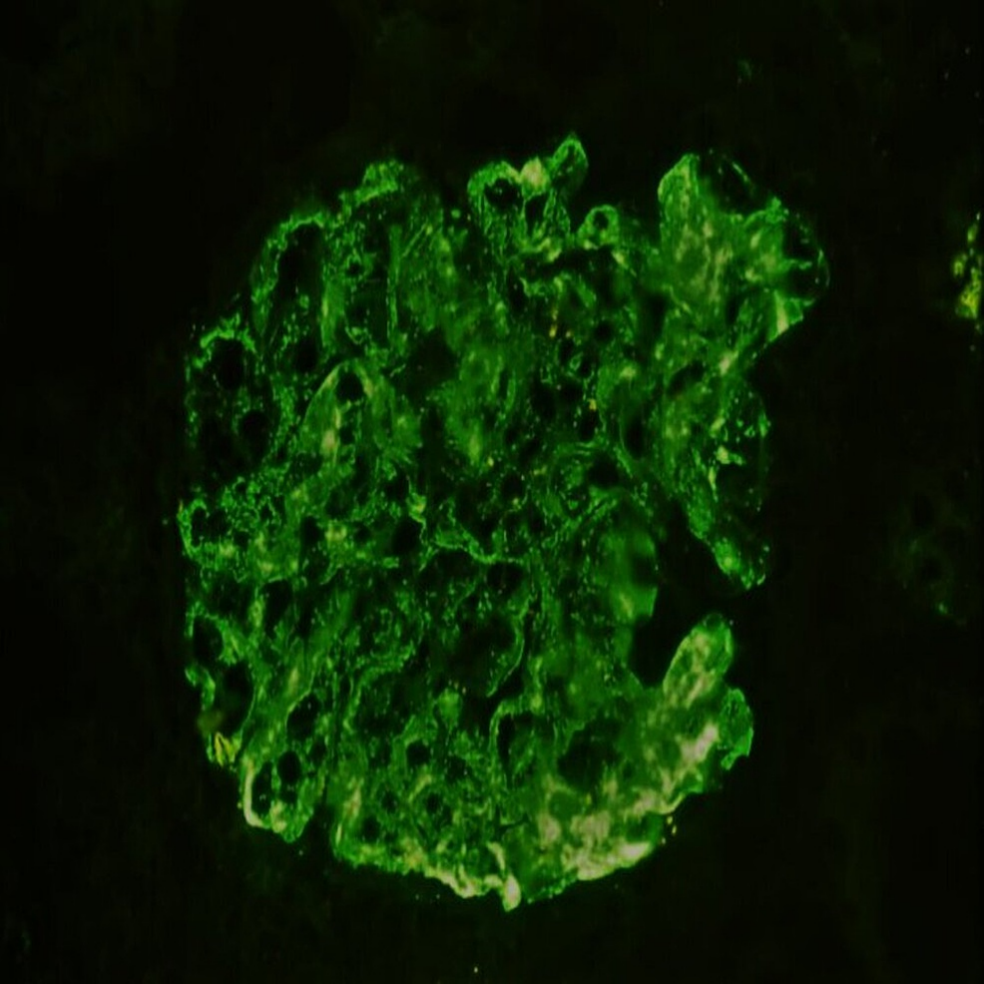
Immunofluorescence showing 3+ intensity mesangial and capillary IgA staining (x200)

**Figure 2:**
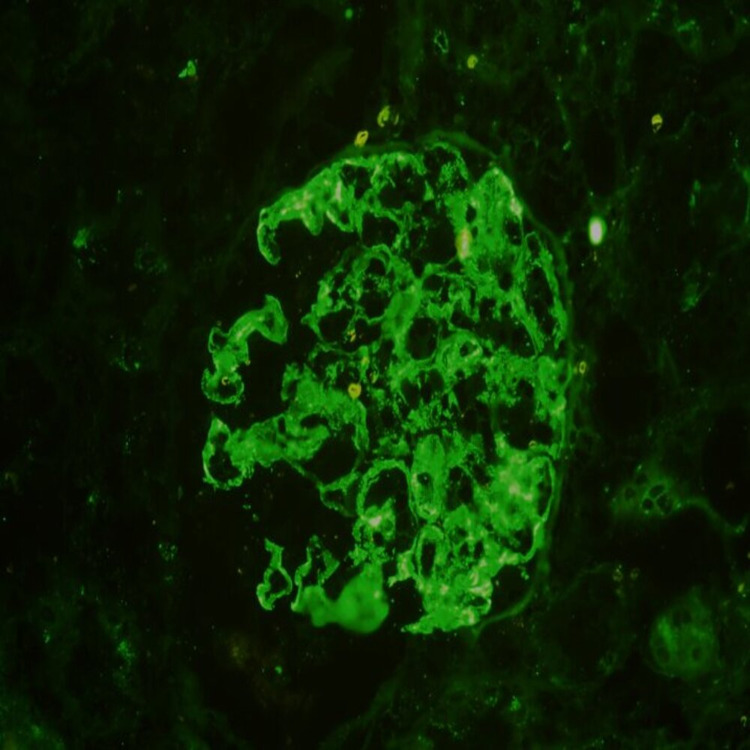
Immunofluorescence showing 3+ mesangial IgG staining (x200)

An ambispective study was carried out over a period of 7.5 years from January 2013 to July 2020 in the Department of Pathology, North Eastern Indira Gandhi Regional Institute of Health and Medical Sciences, Shillong, India. All native pediatric and adult patients with histopathological diagnosis of IgA nephropathy with a minimum of eight glomeruli were included in the study. Native kidney biopsies with the clinical diagnosis of Henoch-Schonlein purpura were excluded from the study. Hematoxylin and eosin stain along with periodic acid-Schiff stain, Masson’s trichrome, and Jones Silver stains was used for histomorphological assessment. Immunofluorescence patterns for IgA and IgG were analysed and all the biopsies were categorized into two groups: non-IgG and IgA plus IgG. Two nephropathologists independently examined the biopsied glomerular tissue for IgA and IgG deposits. When the immunoglobulin deposits were evaluated differently, a consensus was arrived at after discussion between the two nephropathologists. The intensity of the immune deposits was graded on a four-point scale: 0 (absent), 1+ (weak), 2+ (moderate) and 3+ (severe). Segmental mesangial deposit levels (2+ intensity or higher) or global mesangial deposit levels (1+ intensity or higher) were considered as indicative of immunoglobulin deposits. (Figures 1, 2). The location of the immune deposits was also noted [3].

MEST-C scoring was done in accordance with the new 2016 Oxford MEST-C scoring system [6]. Approval for the study was granted by Institute Research and Ethics Committee, North Eastern Indira Gandhi Regional Institute of Health and Medical Sciences (NEIGR/IEC/M7/T10/19).

Age, sex, demographic profile, duration of symptoms of the patient, and presenting symptoms, namely, presence or absence of edema, were noted. If present, edema, whether it was generalized, facial, pedal, facial, or pedal, was noted. Oliguric symptoms of patients were also noted along with the presence or absence of hematuria. Systolic blood pressure (SBP) and diastolic blood pressure (DBP) of the patients were obtained from the medical records. Mean arterial pressure (MAP) was calculated by the following formula: MAP= DBP+1/3 (SBP-DBP).

Amongst the laboratory parameters at presentation, urine analysis for proteinuria was carried out and graded on a scale of 0 to 3+ (nil, mild, moderate, severe) based on the protein strip reading. The estimated glomerular filtration rate (eGFR) was estimated by the Modification of Diet in Renal Disease (MDRD) equation. Patients with an eGFR value ≥90 mL/min/1.73 m^2^ were considered to have a normal kidney function. Serum creatinine levels were analysed by an automated analyzer and values ≥1.1 mg/dL were considered reflective of a poor renal function.

The MEST-C score was analysed in correlation to the clinical as well as laboratory parameters for the IgA and IgG co-dominant cases. All the statistical analysis was done in IBM SPSS Statistics, Version 20 (IBM Corp., Armonk, NY). Chi-square test and independent samples t-test were utilized to correlate various immunofluorescence as well as clinical and laboratory parameters in accordance with the new Oxford MEST-C score system and a probability (p) value <0.05 was considered statistically significant.

## Results

A total of 58 cases of IgA nephropathy fulfilling our inclusion and exclusion criteria were included in the study. Out of 58 cases, 25 biopsies (43.1%) were categorized as IgA plus IgG, and the rest 33 biopsies (56.8%) as a non-IgG group.

The clinical and demographic profiles of the patients in the two categories are summarized in Table 1.

**Table 1:** Analysis of clinical, biochemical, and MEST-C amongst 33 cases (56.8%) of non-IgG immunostain deposition compared to 25 cases (43.1%) with IgA and IgG co-dominance MAP, mean arterial pressure; eGFR, estimated glomerular filtration rate; MEST-C, mesangial hypercellularity, endocapillary hypercellularity, segmental sclerosis, tubular atrophy/interstitial fibrosis, crescents

	Non-IgG (n=33)						IgA plus IgG (n=25)										
Age (years)	Range=2-60	Mean=25.4			SD=13.6		Range=3-66	Mean=21.5			SD=15.05		t=1.02	Df=56		p=0.311	
Gender	Male=18; female=15						Male=06; female=19										
Clinical features	Edema=33; edema and oliguria=12; oliguria=01						Edema=25; edema and oliguria=14; oliguria=00										
Duration (month)	Range=0.25-18		Mean=5.06			SD=5.86	Range=0.25-12		Mean=3.4			SD=3.2	t=1.221		Df=56		p=0.227
Hematuria	Present=18; absent=15						Present=08; absent=17										
MAP (mm of Hg)	Range=70-146.6		Mean=103.4			SD=20.1	Range=80-156.6		Mean=116.2			SD=25.5	t=-2.126		Df=56		p=0.038
Serum creatinine (mg/dL)	Range=0.4-3.9		Mean=1.345			SD=0.81	Range=0.4-4.1		Mean=1.674			SD=1.272	t=-1.197		Df=56		p=0.236
Proteinuria	Mean=2.3			SD=0.769			Mean=2.8			SD=0.374			t=-3.207		Df=56		p=0.002
eGFR (mL/min/1.73m^2^)	N=20-301		Mean=94.8			SD=67.6	Range=17-329		Mean=97.6			SD=97.5	t=-0.129		Df=56		p=0.898
M score	M0=03; M1=30 (91%)						M0=01; M1=24 (96%)										
E score	E0=17; E1=16 (48%)						E0=05; E1=20 (80%)										
S score	S0=18; S1=15 (45%)						S0=14; S1=11 (44%)										
T score	T0=22; T1/T2=11 (33%)						T0=11; T1/T2=14 (56%)										
C score	C0=26; C1/C2=7 (21%)						C0=16; C1/C2=9 (36%)										

In the IgA plus IgG group, one case (1.7%) showed 1+ intensity, 19 cases (32.7%) showed 2+, and 5 cases (8.6%) showed the intensity of 3+ IgG immune deposits, respectively. Out of 25 cases with both IgA and IgG immunofluorescence, 21 cases (84%) showed only mesangial deposits and the rest 4 cases (16%) showed both mesangial and capillary deposits.

A significant correlation was noted amongst the IgA plus IgG group (n=25) with respect to MAP (p=0.038) and proteinuria (p=0.002) as compared to the non-IgG group (n=33) (Table 1).

Correlation of the MEST-C scores of these 25 patients who had IgA plus IgG co-deposition was done with clinical and laboratory parameters. Statistically, a significant correlation was noted for endocapillary hypercellularity with elevated MAP (p=0.04), raised serum creatinine (p=0.005), and decreased eGFR (p=0.002). Similarly, mesangial hypercellularity in combination with endocapillary hypercellularity correlated significantly with elevated MAP (p=0.012), raised serum creatinine (0.001), and decreased eGFR (p=0.001) (Table 2).

**Table 2:** Correlation of MEST-C scores with clinical and laboratory parameters (ME is M1E1; MEST-C means M1E1S1T1/2C1/2) of cases with IgA and IgG co-deposition eGFR, estimated glomerular filtration rate; MAP, mean arterial pressure; MEST-C, mesangial hypercellularity, endocapillary hypercellularity, segmental sclerosis, tubular atrophy/interstitial fibrosis, crescents

n=25	Age	Gender	Edema	Edema and oliguria	Duration	Hematuria	MAP	Serum creatinine	Proteinuria	eGFR
M	1.0	10	1.0	0.440	1.0	1.0	0.36	0.40	0.160	0.360
E	0.64	0.070	1.0	0.133	1.0	1.0	0.040	0.005	1.0	0.002
S	0.69	0.661	1.0	1.0	0.434	0.389	0.677	0.414	0.604	0.677
T	1.0	1.0	1.0	0.116	1.0	1.0	0.115	0.059	1.0	0.115
C	0.41	1.0	1.0	0.677	0.671	0.394	0.088	0.229	1.0	0.401
ME	1.0	0.125	1.0	0.056	1.0	0.624	0.012	0.001	0.234	0.001
MEST-C	1.0	1.0	1.0	0.661	0.630	0.059	0.057	0.051	1.0	0.057

## Discussion

With regard to IgG deposition rates in patients with IgA nephropathy, Haas showed an IgG deposition rate of approximately 45% and Okada et al. showed an IgG deposition rate of 50% along with IgA [7,8]. In the present study, 43.1% of the cases showed IgG co-deposition, which is comparable to the findings of Haas and Okada et al. [7,8].

Amongst the clinical parameters, the mean age at presentation for the IgA plus IgG group was 21.5 years, which was slightly younger as compared to the non-IgG group suggesting a comparatively severe disease. In the present study, IgA plus IgG group showed a female preponderance (76%). Wada et al. also showed similar findings [3].

Asymptomatic microscopic hematuria is the most common clinical feature at presentation for IgA nephropathy [9]. However, in our study edema was the most common clinical symptom at presentation in both the non-IgG group and IgA plus IgG co-dominant group. This finding may be attributed to the absence of a screening program and lack of awareness amongst the patients leading to the progression of the disease.

In the present study, the MAP, serum creatinine levels, and proteinuria ≥3+ at presentation were higher in the IgA plus IgG group in comparison to the non-IgG group showing a poorer renal function at presentation for this group. The IgA plus IgG group had higher MAP and proteinuria at presentation, which was statistically significant, indicating IgG co-deposition as a culprit of declining renal functions. Wada et al. in their study showed a significant proteinuria correlation in IgG co-dominant patients [3].

The new Oxford system for histological scoring of IgA nephropathy introduced in 2016 identified various histopathological variables, namely, mesangial hypercellularity, endocapillary hypercellularity, segmental glomerulosclerosis, tubular atrophy/interstitial fibrosis, crescents as predictors of renal outcome [6]. Bellur et al. in their study noted higher mesangial cellularity and higher endocapillary hypercellularity (57%) in the IgA plus IgG group as compared to the non-IgG group [4]. The presence of IgG was not statistically correlated with focal and segmental glomerulosclerosis, interstitial lesions, and the presence of crescents [4]. In our study, the IgG plus IgA group had higher mesangial hypercellularity, endocapillary hypercellularity, tubular atrophy/Interstitial fibrosis, and crescents. These findings reveal that co-dominant IgG shows a poorer functioning kidney as evident from the light microscopy scoring by the MEST-C scoring system.

We also analysed the MEST-C score variables with the clinical and laboratory parameters amongst the IgA plus IgG group. Statistically, a significant correlation was noted for endocapillary hypercellularity amongst the MEST-C variables with raised MAP, raised serum creatinine level, and a diminished eGFR level at presentation. Thus, these findings suggest that IgA and IgG co-dominant group presents with poorer clinical, histologic, and laboratory findings, which significantly increases the risk for progression to chronic kidney disease in these patients [10].

## Conclusions

The IgG co-dominant group presents with a slightly variable clinical and laboratory picture at presentation. Slightly younger age and female predominance point towards the aggressive nature in the co-dominant cases. The M, E, T, C score was higher in this group. Endocapillary hypercellularity showed a statistically significant correlation with raised MAP, raised serum creatinine level, and decreased eGFR at presentation substantiating the fact that IgG deposition serves as an adverse marker pointing towards a deranged renal function.
